# Pretreatment with licochalcone a enhances therapeutic activity of rat bone marrow mesenchymal stem cells in animal models of colitis

**DOI:** 10.22038/ijbms.2021.56520.12616

**Published:** 2021-08

**Authors:** Meng Chen, Yang Yu, Shiyao Yang, Deqin Yang

**Affiliations:** 1 Department of Endodontics, Stomatological Hospital of Chongqing Medical University, Chongqing, China; 2 Chongqing Key Laboratory of Oral Diseases and Biomedical Sciences, Chongqing, China; 3 Chongqing Municipal Key Laboratory of Oral Biomedical Engineering of Higher Education, Chongqing, China

**Keywords:** Colitis, CXCR4, Inflamation, Licochalcone A, Mesenchymal stem cells

## Abstract

**Objective(s):**

Colitis has a high prevalence rate, limited treatment options, and needs to be solved urgently. Application of Licochacone A (LA) or rBMMSCs alone in the treatment of colitis has a certain but limited effect. This study aims to develop an LA-based strategy to improve mesenchymal stem cells’ (MSCs’) therapeutic capacity in mice DSS-induced colitis by increasing the number of MSCs migrating to the inflammation site.

**Materials and Methods:**

*In vivo*, we injected MSCs pretreated with LA, MSCs alone, or PBS into the tail vein of colitis mice, and assessed the colon length, disease activity index (DAI) score, body weight, HAI score, and tracked the location of MSCs at day 10. *In vitro*, we knocked down the CXCR4 gene by siRNA and then treated it with LA, then tested the mRNA level of CXCR4 and the migration ability of group CXCR4, CXCR4+LA, LA, and control to verify the relationship between this effect and the SDF-1-CXCR4 signaling pathway.

**Results:**

The mice that received LA- pretreated MSCs had ameliorated body weight loss, preserved colon morphology, and decreased DAI and histological activity index (HAI) compared with the MSCs group. Besides, the number of MSCs migrating to the inflammation site signiﬁcantly increased in group LA+MSCs, and expression of CXCR4 signiﬁcantly increased too. Furthermore, we found that LA could partly revise the decrease of the migration of MSCs and the expression of CXCR4 mRNA caused by CXCR4-siRNA.

**Conclusion:**

LA may improve the migration ability of MSCs through increasing CXCR4 expression therapy enhancing their therapeutic activity.

## Introduction

Inflammatory bowel disease (IBD) is a group of unexplained chronic intestinal inflammatory diseases, including ulcerative colitis and Crohn’s disease. IBD affects over 4 million people worldwide. At the turn of the 21st century, it became a global disease with accelerating incidence in Asia (1). The most widely accepted pathogenesis of IBD contains plenty of complicated factors: genetic factors, environmental factors, and intestinal microbiota (2). Several drugs such as TNF blockers, vedolizumab (3), and anti-cytokine agents (4) have been used to treat IBD clinically, but not all patients are suitable for these drugs. Therefore, there is an urgent need for novel therapeutic strategies that are more effective. 

 Mesenchymal stem cells (MSC) are pluripotent stem cells with an immune regulation ability (5). Some studies have reported promising data for the abilities of MSCs in decreasing the expression of various inflammatory cytokines, chemokines, and filtration of inflammatory cells (6). Because of these characteristics, it has been reported that MSCs can be used to treat a series of diseases caused by self-immune responses including multiple sclerosis type diabetes, systemic lupus erythematosus, organ transplantation and concomitant tissue damage, rheumatoid arthritis, and graft-versus-host disease (7). In addition, MSCs can diﬀerentiate into gut epithelial cells and promote angiogenesis. So MSCs are considered a new therapeutic agent in the treatment of IBD (8). MSC can be divided into bone marrow MSC, dental stem cells, adipose stem cells, umbilical cord stem cells, etc., according to their sources. Among them, bone marrow mesenchymal stem cells (BMMSCs) have more extensive sources and relatively simple sources than other cells, considered to be a huge potential (9).

Licochalcone A (LA) is a kind of flavonoid compound extracted from traditional Chinese medicine leguminous licorice (10). Studies have shown that LA has anti-bacterial (11), anti-parasitic (12), anti-tumor (13), and anti-inflammatory (14) characteristics. LA has been frequently used in stomach ulcers, bronchitis, and sore throats (15). It has been reported that LA could relieve symptoms of DSS-induced colitis in mice (16) by inhibiting NF-κB-regulated pro-inflammatory signal transduction and activating Nrf2-regulated cytoprotective protein expression. At present, there is no research on the treatment of human gastrointestinal inflammation with LA, but there are reports on the anti-inflammatory effect of LA on human skin and other parts (14) and other licorice extracts in the treatment of peptic ulcers (15). CXC receptor 4 (CXCR4), located on the cell surface of MSCs, plays an important role in the homing of MSCs to injured sites (17-20) through interaction with its ligand, stromal-derived factor-1 (SDF-1), and expressions of SDF-1 are up-regulated in injured tissues. However, the levels of CXCR4 are down-regulated in MSCs during amplification *in vitro*. 

In this study, we studied the therapeutic effect and molecular mechanism of LA pretreated MSCs on the DSS-induced mouse colitis model. 

## Materials and Methods

***Animals***

96 healthy female eight-week-old C57BL/6 mice and 90 four-week-old Sprague Dawley rats were obtained from the animal center of Chongqing Medical University (Chongqing, China) (21). C57BL/6 mice were used in *in vivo* assay, and SD rats were used in MSC isolation. Mice and rats were living in mice and rat-specific breeding rooms, respectively. All animals had free access to complete nutrient pellet feed and sterilized water. The animal experiment was approved by the Animal Care and Use Committee of Chongqing Medical University. The animal experiment has passed the review of the ethics committee (Research Ethics Committee (REC) reference number is CQHS-REC-2020). 

***Cultivation and identification of MSCs***

Cultivation and identification of MSCs were conducted as previously described (9). In brief, the Sprague-Dawley rats were sacrificed by cervical dislocation. Femurs and tibias were taken under sterile conditions and washed with PBS (Sigma, USA, P3813-10PAK) 3 times on ice. The heads of the femurs and tibia were cut to expose the bone marrow cavity, and then the bone marrow was flushed out using the minimum required medium containing α-MEM (HyClone, USA, SH30265.01), 1% streptomycin/penicillin mixture (Invitrogen, USA, 15070-063), and 10% fetal bovine serum (FBS) (HyClone, USA, SH30396.03) from the purification platform. And cells were grown at 37 °C in a 5% CO_2_ incubator (Thermo Fisher Scientific, USA).

To characterize MSCs, flowcytometry was used to observe the surface markers of the BMMSCs. In brief, the cells were washed with PBS (containing 3% FBS) twice and then diluted into 1×10^6^ cells/ml. The cells were incubated with conjugated monoclonal antibodies against CD90-FITC (BD Bioscience, USA, 561973), CD29-PE (BD Bioscience, USA, 562154), CD45-PE (BD Bioscience, USA, 561867), CD31-PE (BD Bioscience, USA, 555027) at 4 °C in the dark, washed with PBS and then resuspended in 0.5 ml PBS. Then the detection of PE and FITC labeling of MSCs were subjected to ﬂow cytometric analysis (Beckman Coulter, USA). MSCs were seeded onto 6-well plates at a density of 1 × 10^5^ cells/well and cultured in a complete medium containing α-MEM, 10% FBS, and 1% streptomycin/penicillin mixture. For osteogenic differentiation, MSCs were cultured in an osteogenic differentiation medium containing 100 nm dexamethasone (Sigma-Aldrich, USA, D4902), 50 ug/ml ascorbic acid (Sigma-Aldrich, USA, 301361), and 5 mM β-glycerophosphate (Sigma-Aldrich, USA, G9422) for 4 weeks. Then MSCs were stained with 2% Alizarin Red (Sigma-Aldrich, USA, A5533). For adipogenic differentiation, cells were stimulated for 21 days with adipogenic differentiation medium containing 0.5 mM methylisobutylxanthine (Sigma-Aldrich, USA, I5879), 0.5 mM hydrocortisone (Sigma-Aldrich, USA, 3867), and 60 mM indomethacin (Sigma-Aldrich, USA, I7378) and stained with Oil Red O (Sigma-Aldrich, USA, O0625).

***CXCR4 siRNA transfection***

MSCs were seeded in a 6-well plate at a density of 1 × 10^5^ cells/well in a medium containing 10% FBS without antibiotics, 24 hr prior to transfection. SiRNA-CXCR4-1(RioBio, China, siB18031504428), siRNA-CXCR4-2(RioBio, China, siG2101120905123962), and siRNA-CXCR4-3 (RioBio, China, siG2101120905125210) were transfected through Lipofectamine 2000 (Invitrogen, USA, 11668-019) at a final concentration of 50 nM, according to the manufacturer’s instructions on the day of transfection. Incubating them at 37 °C with a 5% CO_2_ atmosphere for 24 hr (22).

***LA pretreatment MSCs***

LA powder (Sigma-Aldrich, USA, 68783) was dissolved with DMSO (MP Biomedicals, USA, 219605580) to a concentration of 10^-4^M and stored in the dark at 4°C, which would be diluted 1000 times with complete medium on the day of pretreating MSCs and be added to the culture dishes or well plates containing MSCs of p3-p5 (23). LA pretreated MSCs could be obtained after 48 hr at 37 °C and a 5% CO_2_ incubator for the following experiments.

***Quantitative real-time polymerase chain reaction assays***

Total RNA was extracted from the MSCs in previous experiments with Trizol (Sigma-Aldrich, USA, 93289) reagent according to manufacturer’s protocol and was reverse-transcribed to cDNA using a PrimeScript RT reagent kit (Takara, Japan, RR037A). SYBR Premix Ex Taq II kit (Takara, Japan, RR820A) and a CFX96TM Real-time RT-PCR System (Bio-Rad, Hercules, CA, USA) were used to perform the real-time RT-PCR analysis. The primer sequences of CXCR4 and β-actin genes used in this study can be seen in Table 1, also the concentration and purity of RNA can be seen in Tables 2 and 3.

***Transwell migration assay***

Cell migration assay was carried out using the Transwell plates (Corning Costar, USA, 3422), which were 6.5 mm in diameter with 8-um pore filters (24). In brief, MSCs were first stained according to the manufacturer’s standard instructions of the PKH26 staining kit. After staining, normal MSCs and LA-pretreated MSCs were carefully suspended seeded into the upper chamber of transwell plates at a density of 1 × 10^6 ^per ml (100 µl per chamber ). 600 µl of the medium was added to the lower chambers. Then the cells were incubated at 37 °C in 5% CO_2_ for 24 hr. MSCs which had penetrated to the bottom side of the membrane were fixed with formalin phosphate for 30 min, and then observed with a fluorescence microscope or observed with an optical microscope after 1% crystal violet-solution staining for 15 min. The number of cells that had migrated to the lower side of the filter was counted in five randomly selected fields. 

***Western blot analysis***

Cells in group MSCs and group LA+MSCs were lysed in lysis buffer (Beyotime, China, P0013B) and proteins were loaded with sodium dodecyl sulfate-polyacrylamide gel (Beyotime, China, P0012AC) and transferred to polyvinylidene fluoride membranes (Beyotime, China, FFP33). Membranes were blocked with 5% nonfat milk powder (Beyotime, China, P0216-1500g) and then probed overnight with CXCR4 (Proteintech, USA11073-2-AP) and β-actin (Abcam, USA, ab8227) primary antibodies. The membranes were incubated with a peroxidase-conjugated secondary antibody (Bioss, China, bs-0293G). The PVDF membrane was treated with an enhanced chemiluminescence kit (Beyotime, China, P0018AS) and subjected to X-ray film, the gray values were measured with Image-Pro Plus 6.0 software (25). 

***Construction of colitis model and rat MSCs tracing***

Refer to Figure 2A for the experiment protocol. In detail, dextran sulfate sodium (DSS) (Sigma-Aldrich, 42867) powder was dissolved with sterile PBS to a 3% (w/v) solution. Mice (C56BL/6) were fed 3% DSS solution for 10 days to induce acute colitis as previously described (26). Animals were randomly divided into four groups: control group (fed with clean water), colitis group, MSCs group (injected MSCs), and LA+MSCs group (injected LA-pretreated MSCs), and all MSCs were stained with PKH26 cell fluorescent staining kit (Sigma-Aldrich, USA, MINI26) before injection (27). On day 3, mice of experimental groups were separately injected 1 × 10^6^ MSCs and the MSCs pretreated by 10^-7^M LA diluted with PBS through tail vein according to a previous study (23), and control and colitis groups were injected equal volumes of PBS. Bodyweight, stool consistency, and presence of fecal blood were recorded daily to score the disease activity index (DAI) (0-4) according to previous research(9). On day 10, mice were sacrificed to get the full colon for length measurement and histopathological analysis, and for observing the number and location of fluorescent cells in each group with a fluorescence microscope for transplanted MSC tracing (Olympus, Japan)(27).

***Histopathological analysis***

Previous colonic segments were fixed in formalin phosphate (Mengbio, China, MBX113) and embedded in paraffin (Solarbio, China, YA0012), then 4 um thick sections were prepared for hematoxylin and eosin (H&E) staining. Histological activity index (HAI) can be used to indicate the degree of pathological changes in the colon tissue and HAI was scored as follows: for epithelium damage, normal morphology was scored as 0, goblet cells lost in limited areas was scored as 1, goblet cells lost in large areas were scored as 2, loss of crypts was scored as 3 and loss of crypts in large areas was scored as 4. For inflammation infiltration, no infiltration was scored as 0; infiltration limited around crypt basis was scored as 1, inflammation infiltration reaching muscularis mucosa was scored as 2, extensive infiltration extending into the muscularis mucosa and thickening of the mucosa with abundant edema was scored as 3, and infiltration of inflammation extending into the submucosa was scored as 4 (28).

***Statistical anlysis***

All experiments were assayed in triplicate. Numerical data are expressed as means ± SD; Data were analyzed using GraphPad Prism software (San Diego, CA, USA) and Microsoft EXCEL version 2003. The student’s t-test was used to compare two groups. One-way analysis of variance (ANOVA) was performed to compare among three or more groups. *P*-values<0.05 were considered statistically significant. 

## Results

***Characterization of MSCs***

**Figure 1:**
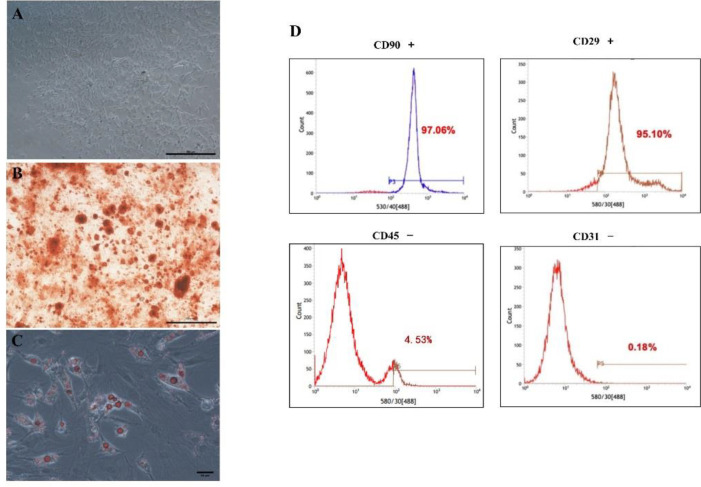
Characterization of Mesenchymal stem cells (MSCs). (A) Morphology (scale bar=100 μm) of MSCs was observed by microscopy. (B) Osteogenic differentiation of MSCs was determined by Alizarin Red S staining (scale bar=100 μm). (C) Adipogenic differentiation of MSCs was observed by Oil Red O staining (scale bar=50 μm). (D) Flow cytometry detected the surface antigens of MSCs, CD 90, and CD 29 were positive; CD 45 and CD 31 were negative

MSCs generated from rat bone marrow were cultured as described above to osteogenesis and adipogenesis. When cultured to passage 3, MSCs colonies showed homogenous spindle-shaped, ﬁbroblast-like morphology and plastic-adherent growth properties, typically (Figure 1A). The osteogenic induction results showed that after 4 weeks of induction, mineralized nodules in MSCs were stained red by Alizarin Red S staining solution (Figure 1B). The results of adipogenic induction showed that after 21 days of culture, intracellular lipid droplets of MSCs in the cytoplasm in adipogenic differentiation culture medium were stained red by Oil Red O staining solution (Figure 1C). The surface antigens of MSCs were detected by flowcytometry, the results demonstrated that MSCs were positive for CD-29 and CD-90 and negative for CD-45, CD-31 (Figure 1D). 

***Clinical symptom analysis of colitis in mice of each group***

**Figure 2:**
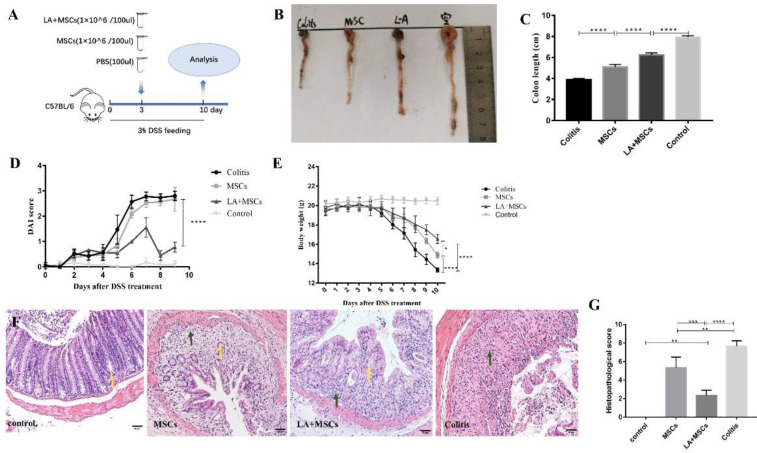
Pretreatment Mesenchymal stem cells (MSCs) with LA attenuated the clinical symptoms of colitis. (A) Schematic diagram showing the experimental protocol. (B) Macroscopic images of colons from each group harvested on day 10. (C) Colon length of each group was measured when the mice were sacrificed. (D) DAI score of each group. (E) Body weight was measured daily during the experimental period. (F) Photomicrographs (scale bar=50 μm) of an H&E-stained paraffin section of a representative mouse colon from each group. Yellow arrows point to goblet cells, dark green arrows point to infiltration of inflammatory cells. (G) HAI of each group. Results were presented as the mean±SD (n=5). *P<0.05; **P<0.01

Clinical manifestation including bodyweight loss, bloody diarrhea, and shortening of the colon associated with colitis was observed in mice of DSS induced colitis. Colon length is associated with the severity of DSS-induced colitis. It was found that colitis caused severe colon shortening, while injection of MSCs reduced the extent of DSS-induced colon shortening, and pretreatment with LA preserved the length of the colon better. (Figures 2B, 2C). The disease activity index (DAI) can quantify the activity of colitis and reflect the activity of colitis more objectively and accurately. It combines the weight loss rate of sick animal, stool consistency, and stool bleeding to score comprehensively. DAI of mice in the colitis group was significantly higher than the negative control group. DAI decreased after injection with MSCs, and the differences in DAI between mice receiving MSCs and LA-pretreated MSCs group were significant (Figure 2D). DSS treatment reduced the body weight, while pretreating MSCs with LA both increased the body weight. Besides, it can be found that pretreating MSCs with LA could maintain the bodyweight better compared with the MSCs group (Figure 2E).

***Histopathological analysis of colitis in mice of each group***

 Histological changes in DSS-induced colitis include the absence of epithelial layer and infiltration of inflammatory cells, which are mainly observed in the distal colon with severity gradually becoming progressively less towards the proximal site, whereas DSS-induced lesions were partially prevented in mice treated with LA-pretreated MSCs (Figure 2F). Histological activity index (HAI) of mice treated with MSCs reduced compared with the colitis group, and LA-pretreated MSCs could reduce HAI more (Figure 2G). The above results indicated that LA-pretreated MSCs could relieve the clinical symptoms and preserve the histological morphology of the colon better than MSCs therapy alone.

***Influence of LA treatments for MSCs migration in vitro and vivo***

**Figure 3:**
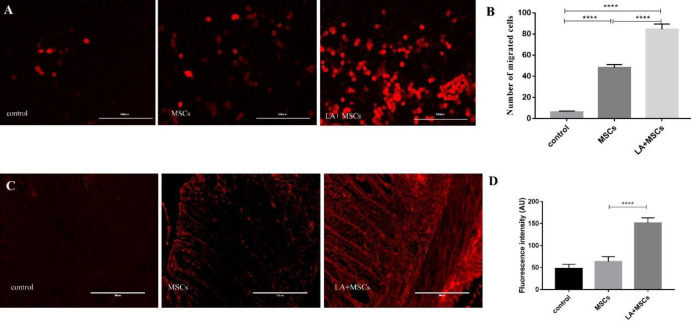
LA improved Mesenchymal stem cells (MSCs) migration *in vitro *and* in vivo*. (A) Photo images (scale bar=400 μm) of matrigel 24 hr after migration experiment *in vitro*. Red fluorescent represented MSCs. (B) Quantification of transwell analysis of cell migration experiment. (C) *Ex vivo* imaging (scale bar=200 μm) of MSCs (PKH26 labeled) in colons. (D)The fluorescence intensity chart of MSCs (PKH26 labeled) of each group. Results were presented as the mean±SD (n=5). *P<0.05; **P<0.01

To track the whereabouts of injected MSCs, cells were labeled with PKH26 cell membrane fluorescent dye before transplantation; a transwell migration assay was conducted to investigate whether LA could elevate the migratory ability of MSCs *in vitro*. The results showed that LA could increase the migratory response of MSCs compared with those in the culture medium without LA (Figures 3A, 3B). At the same time, MSCs (PKH26 labeled) were observed in the colon tissue 7 days after cell transplantation. Among them, the LA+MSCs group showed more cells with red fluorescence than the MSCs group and almost no red fluorescent cells could be seen in the control group (Figures 3C, 3D). The above experiments showed that LA can improve the migration ability of MSCs *in vitro* and to inflamed tissues.

***The effect of LA on MSCs after CXCR4 gene knockdown***

**Figure 4:**
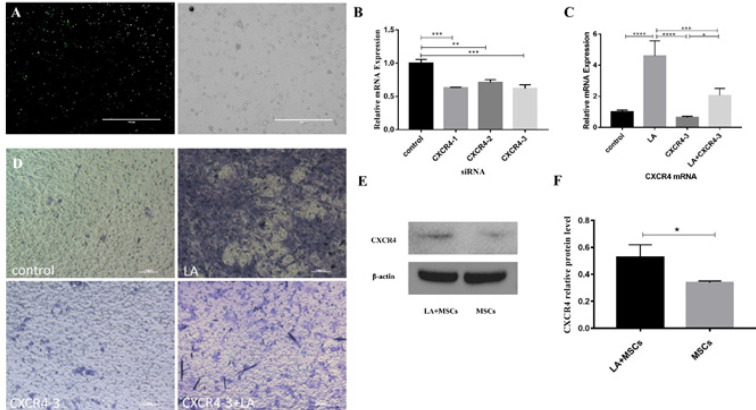
CXCR4 knockdown reduced Mesenchymal stem cells (MSCs) migration, and LA treatment can partially reverse this reduction. (A) Transfection efficiency of CXCR4-siRNA was more than 80%, green fluorescence represents positive cells. (B) CXCR4 mRNA level of MSCs after being transfected with 50 nM CXCR4-specific siRNA (siRNA–CXCR4-1 or siRNA–CXCR4-2 or siRNA–CXCR4 -3) or control siRNA (siRNA–CTRL) for 24 hr. (C) CXCR4 mRNA level of MSCs in each treatment group. (D) Photo images (scale bar=100 μm) of matrigel of each group 24 hr after migration experiment. Purple represented MSCs. (E, F)The protein levels of CXCR4 of MSCs of each group. Results were presented as the mean±SD. (n=5). *P<0.05; **P<0.01

Under the fluorescent microscope, most of the cells were labeled green fluorescence. Five fields of view were randomly selected for statistical analysis, and the transfection efficiency exceeded 80% (Figure 4A). The relative mRNA level of CXCR4 was down-regulated by 39%, 30%, and 37% respectively (Figure 4B) when treated by 50 nM CXCR4-specific siRNA (siRNA-CXCR4-1, siRNA-CXCR4-2, or siRNA-CXCR4-3). After 24 hr of transfecting siRNA-CXCR4-3 into cells, some of them were treated with LA (10^-7^M). Then the RNA of each group (LA+siRNA; siRNA; control) was collected, the mRNA level of CXCR4 in the siRNA group was significantly lower than that of the control group, which proved that the expression of CXCR4 was inhibited by siRNA, and then these cells were treated with LA, the mRNA level of CXCR4 was significantly higher than that of the control group (Figure 4C). MSCs can migrate to injured sites, a transwell experiment verified the change was related to CXCR4 expression of MSCs. The results showed that the number of MSCs that migrated to the lower chamber in LA, LA+CXCR4-3 (MSCs treated with LA after 24 hr transfecting CXCR4 siRNA), negative control, and CXCR4-3 groups decreased successively (Figure 4D), which proved that decreased expression of CXCR4 would reduce the migration ability of cells, and after treatment with an appropriate concentration of LA, the reduction effect could be reversed and the migration effect could be partially restored. Besides, western blot experiments verified expression of CXCR4 protein was significantly elevated in MSCs pretreated with LA (Figures 4E, 4F), These results suggested that LA can partially reverse the reduction of migration caused by CXCR4 knockdown.

## Discussion

In this study, we clearly demonstrated that LA effectively promotes expression of the CXCR4 gene of BMMSCs, thereby promoting the migration of BMMSCs, and it reduced colitis inflammation in mice after injecting LA-pretreated rBMMSCs.

**Figure 5:**
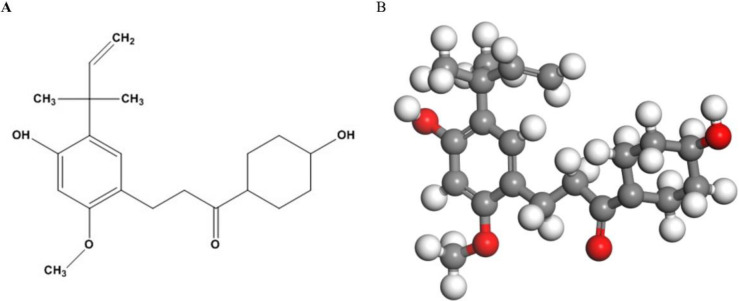
Chemical structure of licochalcone A. (A) the structural formula. (B) the chemical structure image

IBD is a multifactorial relapsing disease of the gastrointestinal tract. MSCs are an important source for treatment of chronic inflammatory diseases because they can be widely isolated and have low immunogenicity and immunomodulatory capabilities (29, 30). A few studies have shown that MSC transplantation is a promising treatment strategy for IBD (31). Among them, BMMSCs are a kind of ideal stem cells due to their special biological characteristics, such as immune regulation ability and being relatively easy to get (32, 33), thus we decided to use BMMSCs in this study. However, it is necessary to strengthen the therapeutic effect of BMMSCs (34). LA is a characteristic chalcone (Figure 5) found in licorice root which has anti-inflammatory properties. It has been found that LA is effective in preventing DSS-induced colitis through inhibiting NF-κB-regulated pro-inflammatory signaling and activating Nrf2-regulated cytoprotective protein expression (16). In this experiment, we determined that MSCs pretreated with LA rather than MSCs alone had a better therapeutic effect on DSS-induced colitis in mice, which might be due to the migratory ability changes of MSCs after LA treatment. Our results showed that MSCs could relieve inflammation symptoms and repair damaged tissues. The MSCs pretreated with LA showed a better curative effect and had improved colitis therapeutic effect in mice. 

Many studies have confirmed the anti-inflammatory effect of LA, and the research on its anti-inflammatory mechanism is mainly on the influence of LA on inflammation-related signal pathways, such as NF-κB-regulated pro-inflammatory signaling (16) and MAPK signaling pathways (35). In this study, we found that MSCs treated with LA migrated more to the site of inflammation of the colon to participate in tissue repair and improved the therapeutic effects. Many studies have shown that MSCs can migrate to various parts of tissue damage and inflammation; this feature is an important way for its therapeutic efficacy (36, 37). However, some studies have found that the efficiency of MSCs migration and homing to inflamed tissues is a bit low (38, 39). CXCR4 is a seven-transmembrane G-protein-coupled receptor, whose activation leads to intracellular signaling cascades (40). Plenty of studies have found that SDF-1 expresses in a variety of tissues and increases its expression in injured tissues (41), and CXCR4 is a specific receptor for SDF-1 (42). It is known that expression of CXCR4 is involved in the migration and homing process of MSCs. However, due to decreased CXCR4 expression during *in vitro* expansion, the homing ability of MSCs is reduced (43). Therefore, increasing the expression of CXCR4 is vital to improve the homing efficiency of MSCs. In order to explore the mechanism of enhanced migration ability of LA-pretreated MSCs, we conducted a series of *in vitro* experiments. Firstly, western blot experiments verified expression of the CXCR4 protein was significantly elevated in MSCs pretreated with LA. Then, we knocked down the CXCR4 gene and found siRNA-CXCR4 transfection could effectively reduce the expression of CXCR4 in MSCs and the migration ability of MSCs decreased with decline of CXCR4 expression, and then, we treated those MSCs with LA and found LA could reverse this down-regulation and partially restore the expression of CXCR4 and partially improve the decline of the migration ability caused by the decrease of CXCR4 gene expression. Considering CXCR4 activation can lead to intracellular signaling cascades and SDF-1/CXCR4 is a classic signal pathway for cell migration, we suppose that LA may promote the therapeutic effect of MSCs through the signal pathway, and we have verified the effects of CXCR4. However, the detailed information about the LA-SDF-1/CXCR4 signal pathway still needs further testing. At present, there is no relevant research on the chemical structure of LA molecules that affect these therapeutic effects. This also requires to be further explored in the future.

**Table 1:** Primer sequences

**Gene symbols**	**Primer Sequences (5 to 3)** **Upper strand: sense** **Lower strand: antisense**	**Annealing (0 ** **°** **C)**	**Cycle**	**Reference**
CXCR4	ATCTGTGACCGCCTTTACCC CAGGACAGGATGACGATGCC	60	37	（^20^）
**β-actin**	**TGGCACCCAGCACAATGA** **A** **CTAAGTCATAGTCCGCCTAGAAGCA**	**6** **0**	**3** **7**	**（** 22 **）**

**Table 2:** Concentration and purity of RNA

**S** **ample**	**RNA concentration (ng/** **μ** **l)**	**RNA purity (** **λ** **260/** **λ** **280 nm)**
control	1830.3	2.01
CXCR4-1	1827.4	2.00
CXCR4-2	2207.8	1.97
**C** **XCR4-3**	**1** **635.9**	**2** **.02**

**Table 3:** Concentration and Purity of RNA

**S** **ample**	**RNA concentration (ng/mL)**	**RNA purity (** **λ** **260/** **λ** **280 nm)**
control	1689.7	2.00
LA	1734.6	2.03
CXCR4-3	1846.2	1.96
**LA+** **C** **XCR4-3**	**1** **876.9**	**2** **.00**

## Conclusion

LA pretreatment of MSCs can improve the migration ability of MSCs by increasing the expression of CXCR4, thereby improving the therapeutic effect of DSS-induced colitis mice. The number of MSC migrating to the inflammation site may have an impact on the therapeutic effect, but the in-depth mechanism still needs to be further explored.
